# The p11/S100A10 Light Chain of Annexin A2 Is Dispensable for Annexin A2 Association to Endosomes and Functions in Endosomal Transport

**DOI:** 10.1371/journal.pone.0001118

**Published:** 2007-10-31

**Authors:** Etienne Morel, Jean Gruenberg

**Affiliations:** Department of Biochemistry, University of Geneva, Geneva, Switzerland; University of California at Berkeley, United States of America

## Abstract

**Background:**

Annexin A2 is a peripheral membrane protein that belongs to the annexin family of Ca^2+^ and phospholipid-binding proteins. This protein, which plays a role in membrane organization and dynamics in particular along the endocytic pathway, exists as a heterotetrameric complex, consisting of two annexin A2 molecules bound via their N-termini to a dimer of p11/S100A10 light chains. The light chain, and thus presumably formation of the heterotetramer, was reported to control annexin A2 association to the plasma membrane and to cortical actin, as well as the distribution of recycling endosomes. However, the specific role of the light chain and the functions of monomeric versus heterotetrameric annexin A2 have remained elusive in the endocytic pathway.

**Methodology/Principal Findings:**

Here, we have investigated whether p11 plays a role in the endosomal functions of annexin A2. Using morphological and biochemical approaches, we found that p11, unlike annexin A2, was not present on early endosomes. Neither was the heterotetramer detected on purified early endosomes, while it was clearly present in total cell lysates. Moreover, knockdown of p11 with siRNAs did not affect annexin A2 targeting to early endosomes, and, conversely, binding of annexin A2 to purified endosomes or liposomes occurred without p11 *in vitro*. Finally, while we could confirm that annexin A2 knockdown inhibits transport beyond early endosomes, p11 knockdown had no such effects on early-to-late endosome transport.

**Conclusions/Significance:**

Our data show that the binding of annexin A2 to endosomal membranes and its role in endosomal trafficking are independent of the p11/S100A10 light chain. We thus conclude that annexin A2 functions are fully supported by the monomeric form of the protein, at least the endocytic pathway leading to lysosomes.

## Introduction

Annexins form a family of peripheral membrane proteins that can coordinate Ca^2+^ ions via conserved α-helical repeats (annexin repeats). Most members of this family also share the capacity to bind negatively charged phospholipids and thus membranes, and are believed to play a role in membrane organization and dynamics, perhaps as scaffold proteins [1], [2], [3]. Like the majority of annexins, annexin A2 (AnxA2) is composed of a conserved C-terminus core domain folded *via* annexin repeats and a hypervariable non-folded N-terminus. AnxA2 N-terminal domain is small (24 amino acids) and bears two putative phosphorylation sites Tyr23 and Ser25, which are presumably targets of Src kinase and protein kinase C, respectively [4], [5], [6], as well as the binding site for its natural ligand p11/S100A10 [2], [7], [8]. Interactions of two molecules of AnxA2 with two molecules of p11 lead to the formation of the (AnxA2)_2_-(p11)_2_ heterotetramer [9]. It has been reported that the p11 light chain is required for AnxA2 binding to the plasma membrane and to the cortical actin network [10], both mechanisms being also regulated by the presence of Ca^2+^ [2], [3]. Evidence also suggests that the (AnxA2)_2_-(p11)_2_ heterotetramer plays a role in the subcellular distribution of early and recycling endosomes [11], [12] and in the channel functions of cystic fibrosis conductance regulator protein CFTR [13].

AnxA2 has been shown to play a crucial role at early stages of the endocytic pathway by participating to both the recycling pathway [12] and the degradation pathway leading to late endosomes and lysosomes [14], [15]. The protein is present on early endosomes [16], but, unlike other members of this protein family, membrane association does not depend on calcium ions [16], [17], but on membrane cholesterol [14], [15], [18], suggesting that AnxA2 binds to or participates in the formation of cholesterol-rich platforms on endosomal membranes. Moreover, this Ca^2+^-independent endosomal localization depends on the small hypervariable N-terminal domain of AnxA2 [15], [17], [18], [19] which also contains not only phosphorylation sites but the p11 binding region.

In the present study, we have investigated the putative role of the p11 light chain in AnxA2 association to early endosomes and in endosomal trafficking. We report that, in contrast to AnxA2, p11 is not present on early endosomes, and that the (anxA2)_2_-(p11)_2_ heterotetramer is not detected on purified endosomes. Moreover, we find that silencing p11 expression does not affect AnxA2 targeting to early endosomes *in vivo*. Consistently, AnxA2 binding to liposomes or purified endosomes occurs without p11 *in vitro*. Finally we show that downregulation of p11 has no effect on transport from early to late endosome — in marked contrast to AnxA2 downregulation [15]. We conclude that AnxA2 functions are fully supported by the monomeric form of the protein in the endocytic pathway leading to lysosomes.

## Results

### Morphological analysis of p11 subcellular distribution

**Figure 1:**
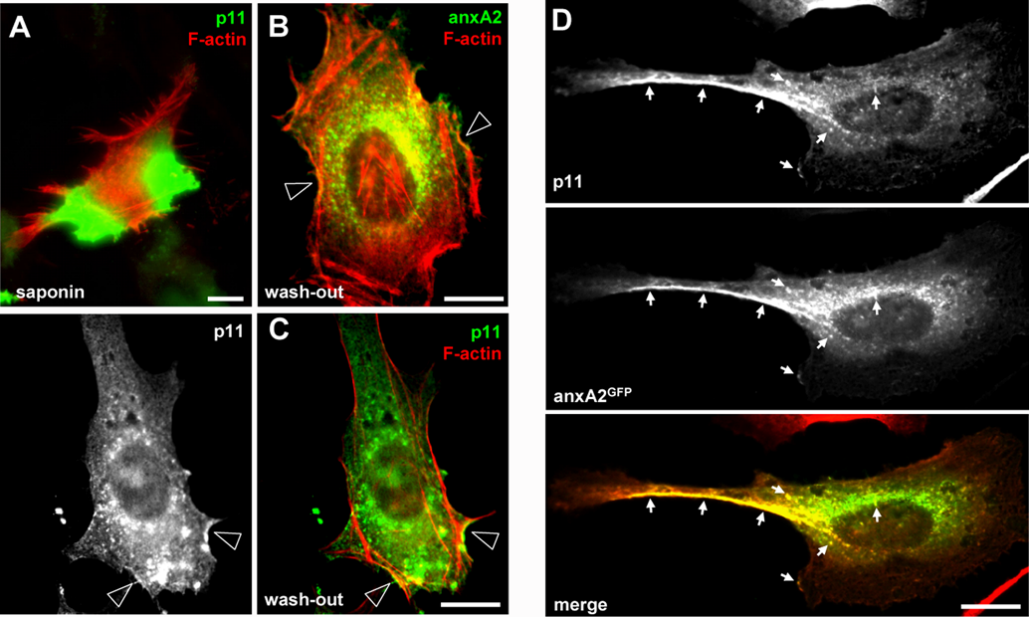
Subcellular localization of p11 compared to F-actin and annexin A2. The distribution in HeLa cells of p11 (H21 monoclonal antibody), AnxA2 (HH7 monoclonal antibody, and F-actin (phalloidin) was analyzed by immunofluorescence using a conventional saponin-based permeabilization protocol (A) or a protocol optimal for AnxA2 and p11 detection after cytosol wash-out (B–D), as follows: (A) p11 and F-actin double fluorescence after saponin-based permeabilization; (B) AnxA2 and F-actin double fluorescence after cytosol wash-out; (C) p11 and F-actin double fluorescence analysis after cytosol wash-out; (D) p11 and AnxA2-GFP double fluorescence analysis after cytosol wash-out. Arrowheads in B and C indicate concentrated AnxA2 and/or p11 bundles at cell surface, with strong colocalization with F-actin. Arrows in D indicate p11/AnxA2 colocalization. Bar: 10 µm.

**Figure 2:**
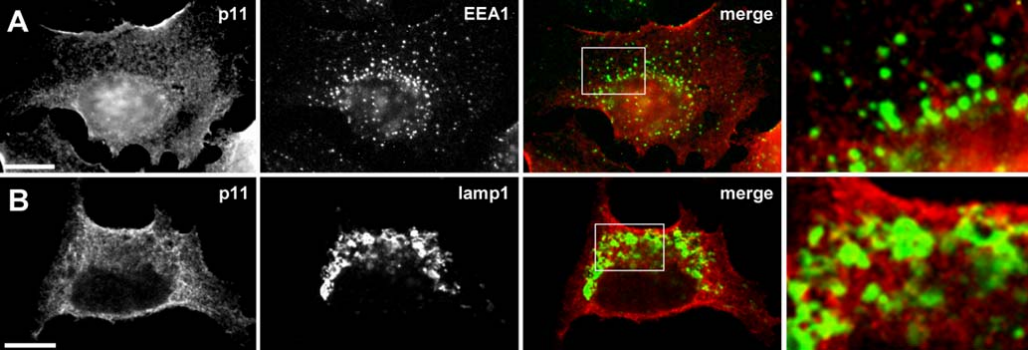
p11 does not colocalise with endosomal markers. The distribution of p11 was analyzed as in Fig 1 (washout protocol), using antibodies against EEA1 (A) and Lamp1 (B). Since AnxA2 is present on early endosomes, we used wide field, and not confocal, microscopy in (A) to ensure that structures that may contain both p11 and the early endosomal marker EEA1 were not missed in this analysis (hence some background nuclear staining). Cropped images are shown on both right panels. Bar: 10 µm.

To investigate the putative p11-AnxA2 interplay, we first compared p11 subcellular localization in HeLa cells to that of anxA2 by light microscopy. To this end, we used a special triton-based permeabilization buffer that allowed better p11 detection and analysis by immunofluorescence (Fig 1C, see methods), when compared to the more conventional saponin-based protocol (Fig 1A). Then, p11 showed a reticulo-vesicular pattern (Fig 1C, left and right panels) and often appeared concentrated, like AnxA2 (Fig 1B), at sites where F-actin patches were observed at, or close to, the cell surface (arrowheads in Fig 1C). Consistently, p11 and AnxA2 showed extensive colocalization at the plasma membrane and in structures reminiscent of actin-positive structures (Fig 1D, see arrows). However, in marked contrast to AnxA2 [15], [16] (see Fig 5D), p11 was not detected on early endosome labeled with antibodies against EEA1 (Fig 2A), an effector of the small GTPase Rab5. As expected, p11 also failed to colocalize with the late endocytic marker Lamp1, a trans-membrane glycoprotein (Fig 2B). We thus concluded that p11 could not be detected on early or late endosomes.

### Biochemical analysis of p11 distribution

**Figure 3:**
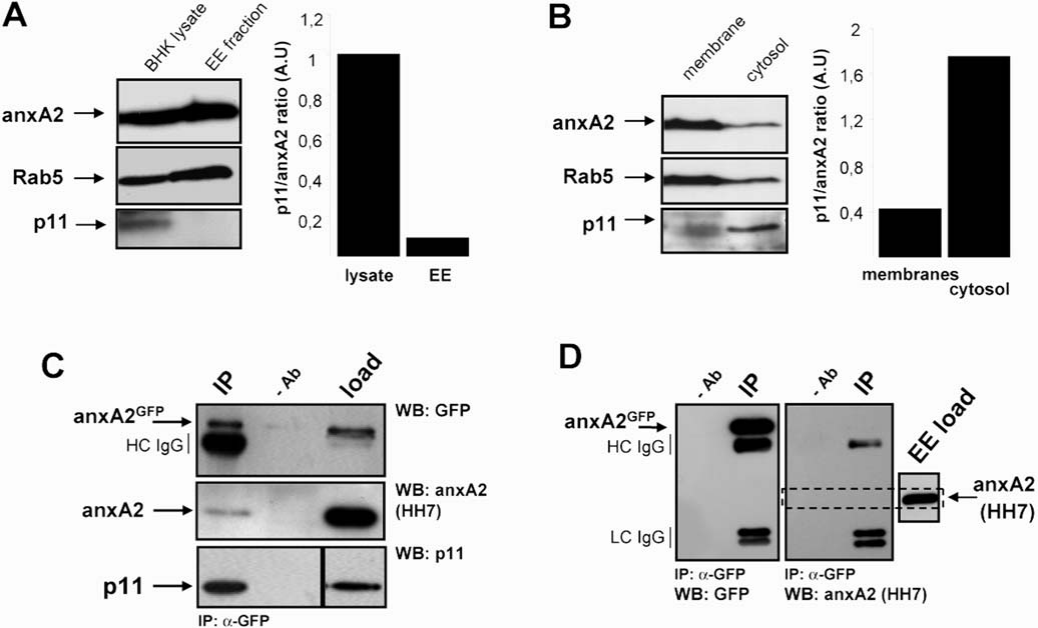
Biochemical analysis of p11 distribution and annexin A2/p11 interaction detection. (A), Total cell lysates and early endosomes (EE) were prepared from BHK cells, and then analyzed by SDS gels (equal proteins amounts loaded in each lane) and western blotting using the indicated antibodies. The p11 and AnxA2 signals were scanned and the right panel shows the ratio of the p11 over AnxA2 signals in the corresponding fractions (A.U: arbitrary units). (B) Experiments and quantification (right panel) were as in (A) except that total membranes and cytosol obtained after high speed centrifugation were analyzed. (C) Hela cells were transfected with (AnxA2^H28^-GFP), and cell lysates were prepared. Then, AnxA2-GFP was immunoprecipitated from the lysates with anti-GFP antibodies (IP: immunoprecipitate; Ab- : control without the specific antibody; load: input fraction before immunoprecipitation). Samples were then analyzed by SDS gels and western blotting with the indicated antibodies. The H28 antibody only recognizes AnxA2^H28^, while, in our hands, the HH7 antibody recognizes WT endogenous AnxA2. HC IgG, heavy chain of anti-GFP antibody used for immunoprecipitation. (D) The experiments were as in (C), except that purified early endosomes were used as starting materials. The left panel shows western blots with anti-GFP antibody, while the right panels show blots with anti-AnxA2 antibody. For comparison, the small right panel (EE load) indicates the mobility of (untagged) AnxA2 in a gel of the early endosome (EE) starting material (load). HC IgG, heavy chain of anti-GFP antibody used for immunoprecipitation, LC IgG, light chain of anti-GFP antibody.

We analyzed p11 distribution biochemically, using a well-established protocol for the fractionation of endosomes from BHK cells (see methods [20]). While p11 was abundant in the total cell lysate, the protein could not be detected in purified early endosomal fractions containing AnxA2 and Rab5 (Fig 3A, left panel). Quantification of the western blot signals from three independent experiments showed that the p11 over AnxA2 ratio dropped in early endosomes to approximately 1/10^th^ of the value measured in total lysates (Fig 3A, right panel). By contrast, the p11 over AnxA2 ratio increased approximately 5 fold in cytosol when compared to total membranes, after high speed centrifugation. AnxA2 and Rab5 co-fractionated predominantly with membranes, as expected (Fig 3B). These observations led us to conclude that p11 does not cofractionate with early endosomal membranes.

**Figure 4:**
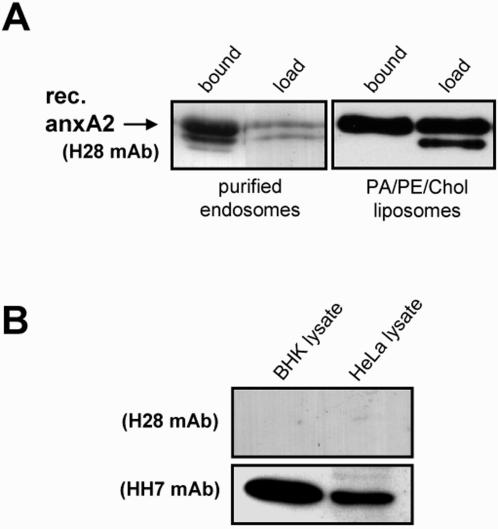
Recombinant annexin A2 binding to endosome and liposome membranes. (A) Early endosomes (left panel) or PA/PE/cholesterol liposomes (right panel) were prepared and incubated in vitro for 30min at 37°C with 5 µg purified recombinant AnxA2 (AnxA2^H28^). Membrane-bound and free AnxA2 were then separately recovered after high speed centrifugation, and analyzed by SDS gels and western blotting, as indicated. The faster migrating form of AnxA2 in panel A corresponds to the core domain of the protein lacking the N-terminus, and results from AnxA2 proteolytic cleavage. Note that the core domain does not become efficiently membrane-associated, as expected [15], (B) The recombinant mutant AnxA2^H28^ is selectively detected with the monoclonal antibody H28 (see 4A), which does not detect WT AnxA2 [23], while the HH7 monoclonal antibody recognizes WT AnxA2 [10], as shown with blots of HeLa and BHK lysates.

We then investigated whether the AnxA2-p11 complex could be retrieved from endosomes by immunoprecipitation. For this purpose, we used human AnxA2, but with the alanine residue at position 65 replaced with an aspartic acid, which is found at the corresponding position in the porcine sequence [21]. The A65E mutant is only detected by the H28 anti-AnxA2 antibody, and will be referred to in the paper as AnxA2^H28^. Additionally, the HH7 monoclonal antibody recognizes selectively endogenous AnxA2 in HeLa and BHK cells ([10], [21], [22], [23] and Fig 4B) but fails, in our hands, to detect recombinant or GFP-tagged anxA2^H28^. When expressed in HeLa cells, GFP-AnxA2^H28^ was properly targeted to early endosomes (Fig 3D, 5D). Then, p11 was efficiently co-immunoprecipitated together with AnxA2^H28^-GFP from total cell lysates, using anti-GFP antibody (Fig 3C), further demonstrating that the A65E mutation did not affect the AnxA2 p11 binding site. Similarly, endogenous AnxA2 was also co-immunoprecipitated with AnxA2^H28^-GFP (Fig 3C), demonstrating that endogenous AnxA2 copurified with both AnxA2^H28^-GFP and p11 within the same complex, presumably as a (AnxA2^H28^-GFP)-(AnxA2)-(p11)_2_ heterotetramer. These immunoprecipitation experiments were then carried out using early endosomes purified from BHK cells expressing AnxA2^H28^-GFP. While AnxA2^H28^-GFP was efficiently immunoprecipiated from purified endosomes, p11 could not be detected in the early endosomal fractions used as starting materials as expected (Fig 3A), let alone in the immunoprecipitates (Fig 3D). Neither was endogenous AnxA2 (Fig 3D), suggesting that endosomal AnxA2^H28^-GFP was monomeric and not complexed to other AnxA2 molecules as a heterotetramer. Altogether these observations indicate that p11 cannot be detected on endosomes biochemically or morphologically.

### Interactions of monomeric annexin A2 with endosomes and liposomes

Since our immunoprecipitations experiments suggested that monomeric AnxA2 was present on endosomes, we tested whether the monomer indeed had the capacity to bind endosomal membranes *in vitro*. Early endosomal fractions prepared from BHK cells were incubated for 30 minutes at 37°C with purified recombinant human AnxA2^H28^ in the absence of cytosol, and then endosome-associated AnxA2 was separated from free AnxA2 by sedimentation (see methods). Recombinant (AnxA2^H28^) and endogenous AnxA2 could then be detected selectively using the H28 and HH7 antibodies, respectively (Fig 4A–B and [10], [23]). This analysis demonstrated that monomeric AnxA2 could bind early endosomal membranes with high efficiency (Fig 4A left panel). Since purified early endosomes contain endogenous AnxA2, one may envision that the exogenously added recombinant AnxA2^H28^ interacted with endogenous AnxA2 present on endosomes. However, monomeric AnxA2^H28^ also associated with high efficiency to PA:PE:cholesterol liposomes (Fig 4A, right panel), which can fully support AnxA2 binding *in vitro* [15]. These experiments thus demonstrate that monomeric AnxA2 exhibits the intrinsic capacity to bind endosome and liposome membranes in the absence of the light chain p11.

### Endosomal targeting of annexin A2 is not modified after p11 knockdown

**Figure 5:**
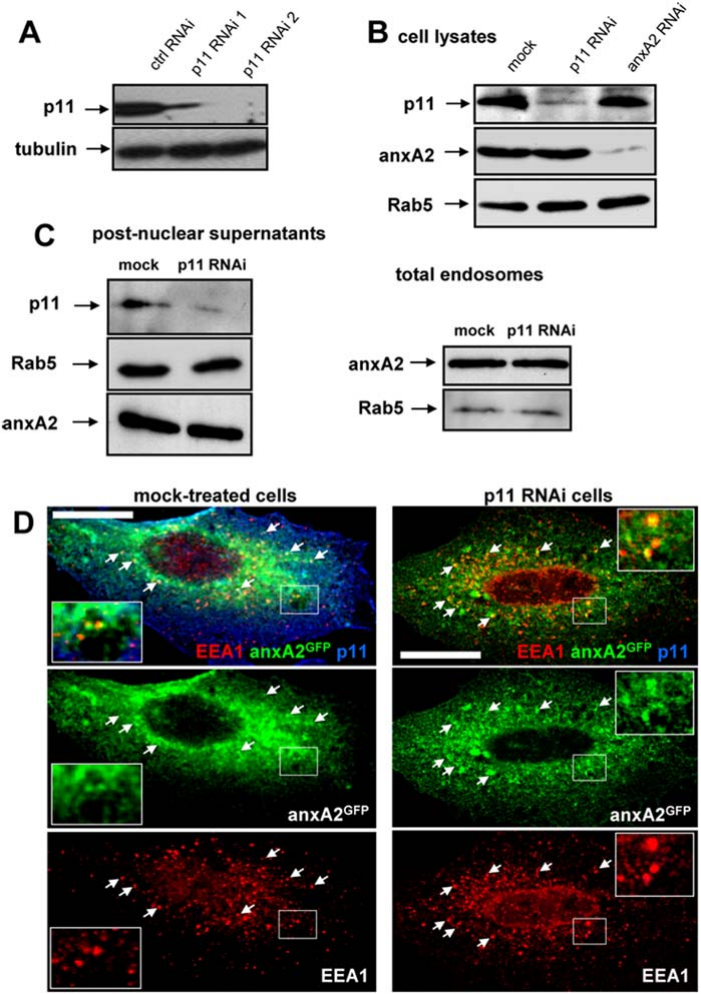
Endosomal targeting of annexin A2 is not affected when p11 is downregulated. (A) p11 was downregulated in HeLa cells using two siRNA duplexes (RNAi1 and RNAi2), and lysates were prepared. The control (ctrl RNAi) siRNA used was designed against viral stomatitis virus (VSV-G) G-protein, which is not expressed in HeLa cells; equal amount of proteins were loaded in each lane and tubulin was used as an equal loading marker. (B) AnxA2 (diced AnxA2 RNAi) or p11 (RNAi2) was downregulated in HeLa cells, and total lysates were analyzed by western blotting as indicated (C) p11 was knocked down (as in A–B), and then a post-nuclear supernatant was prepared (left panel) and used as to further fractionate total endosomes (right panel). The samples were then analyzed by western blotting using the indicated antibodies. (D) AnxA2-GFP (AnxA2^H28^-GFP) was expressed in mock-treated (left panel) or p11-siRNA2-treated HeLa cells (right panel) and then cells were processed for immunofluorescence as in Fig 1 (using the special permeabilizaiton protocol). Arrows show AnxA2-GFP/EEA1 colocalizations and cropped images are shown under both conditions. Bar: 10 µm.

Since monomeric AnxA2 efficiently bound membranes in the absence of p11 *in vitro*, we then investigated whether AnxA2 was properly targeted to endosomes *in vivo*, when p11 expression was silenced. Knockdown of p11 in HeLa cells could be achieved with either one of two siRNAs, siRNA2 being more efficient (Fig 5A). Downexpression of p11 with siRNA2 had no effect on the total cellular amounts of AnxA2 or Rab5 (Fig 5B). Conversely, efficient downexpression of AnxA2 using Dicer-generated siRNAs did not affect p11 expression (Fig 5B). It is, however, possible that p11 silencing interfered with the capacity of AnxA2 to interact with membranes, without affecting its level of expression. We thus prepared post-nuclear supernatants (PNS) from mock-treated or p11 siRNA2-treated HeLa cells (Fig 5C left panel), which were then further fractionated by floatation in gradients to purify membrane fractions containing both early and late endosomes (Fig 5C right panel), [24]. No difference in the amount of membrane-associated AnxA2 was observed when comparing total endosomal fractions from mock-treated or p11 siRNA2-treated HeLa cells (Fig 5C right panel). When mock-treated cells were analyzed by immunofluorescence, AnxA2 showed a characteristic endosome-like punctate pattern and colocalized extensively with EEA1, consistently with its distribution to early endosomes [15], [16] (Fig 5D, left panel, arrowheads). After p11 knockdown (Fig 5D, right panel, arrowheads), we did not observe any difference in AnxA2 distribution and in the colocalization of AnxA2 with EEA1 (quantification shows that the number of EEA1-positive vesicles that contained AnxA2 after p11 knockdown was essentially identical to the mock-treated control, corresponding to 104%±1.4). Altogether these data show that p11 silencing has no effect on anxA2-targeting to early endosomes.

### Early to late endosomal transport is not affected by the absence of p11

**Figure 6:**
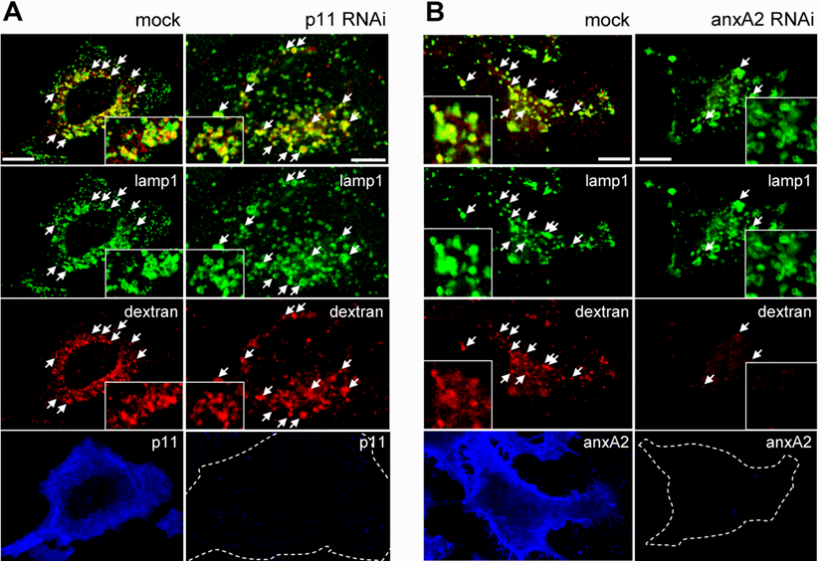
p11 downregulation does not alter transport from early to late endosomes. Rhodamin-dextran was endocytosed for 10 min at 37°C and then chased for 40 min in marker-free medium, after p11 knockdown as in Fig 5 (left panels, with the corresponding mock-treatment on the left) or AnxA2 knockdown as in Fig 5 (right panels, with the corresponding mock-treatment on the left). Cells were then processed for immunofluorescence using the indicated antibodies as in Fig 1. Bar: 10 µm.

In previous studies, we found that AnxA2 knockdown selectively inhibits the transport of endocytosed tracers from early to late endosomes: newly-formed multivesicular endosomes do not detach from early endosomes, and thus fail to mediate transport towards late endosomes [15]. We thus investigated whether p11 was required to support these functions of AnxA2 in endosomal transport. When mock-treated HeLa cells were incubated with rhodamin-dextran for 10min at 37°C, the tracer reached early endosomes containing AnxA2, EEA1 and Rab5 (not shown) [15], [25], [26]. After a subsequent 40min incubation in marker-free medium, the tracer reached late endosomes containing Lamp1 (Fig 6A–B, left panels), as expected [15], [25], [26]. Knockdown of AnxA2 had no effect on rhodamin-dextran transport to early endosomes (not shown), consistently with our previous observations [15]. After the chase, however, rhodamine-dextran failed to reach Lamp1-positive late endosomes and was no longer detected intracellularly in cells lacking AnxA2 (Fig 6B, right panels; the number of vesicles containing Lamp1 and dextran was reduced to 7.2%±1 of the mock-treated control after AnxA2 knockdown). Most likely, the tracer had then been recycled to the medium, rather than transported to late endosomes and lysosomes [15]. Hence, early-to-late endosome transport was inhibited by AnxA2 knockdown, while internalization and recycling did not appear to be significantly affected, as shown previously [15]. Downexpression of p11 did not affect the internalization of rhodamin-dextran into early endosomes (not shown), much like in mock-treated cells or cells lacking AnxA2. However, in marked contrast to the fate of the tracer in AnxA2-silenced cells, rhodamin-dextran was transported after the chase to Lamp1-positive late endosomes in p11 knockdown cells as efficiently as in mock-treated cells (Fig 6A, right panels, the number of vesicles containing Lamp1 and dextran after p11 knockdown was essentially identical to the mock-treated control corresponding, corresponding to ≈94%±1). We thus conclude that AnxA2 functions in membrane transport from early to late endosomes are independent of the light chain p11.

## Discussion

Members of the annexins family are believed to be involved in a wide range of biological functions and the specificity of these functions can presumably be determined by proper targeting to the correct membrane or membrane domain. The AnxA2/p11 heterotetramer is believed to play a role in exocytosis [27], and at the plasma membrane in membrane scaffolding during macropinocytosis [28] and junctional platforms formation [29]. Moreover, in addition to its functions in the degradation pathway leading to late endosomes and lysosomes [15] (and this study), AnxA2 also plays a role in the recycling pathway, perhaps as a heterotetramer [12]. In addition, it has also been suggested that annexin A1, which is closely related to AnxA2, is targeted to early endosomes with its light chain p10/S100C/S100A11, most probably as a tetrameric complex [30].

Here, we demonstrate that AnxA2 functions in endosomal membrane transport do not depend on the light chain p11. Previous studies from others [17], [19] and us [15], [18] have shown that AnxA2 association to early endosomes does not depend on calcium ions but on membrane cholesterol, and requires the small N-terminal domain of the protein, which contains the p11 binding site and the phosphorylation sites. This is further illustrated by the fact that the core domain alone (lacking the N-terminus) does not bind bilayers in the absence of calcium [15]. One might thus envision that the N-terminal region of AnxA2 cannot accommodate p11 spatially while fulfilling the endosomal functions of the protein. The short AnxA2 N-terminus may form an amphipathic helix [31], [32], and structural studies revealed that p11 binds an N-terminal AnxA2 peptide through hydrophobic interactions [9]. It is thus conceivable that, in the absence of p11, the N-terminal amphipathic helix dips into the bilayer [33] and interacts with lipid tails and perhaps cholesterol itself.

Future work will clearly be necessary to determine why the p11 light chain seems dispensable for AnxA2 functions in the degradation pathway (this study), while the (AnxA2)_2_-(p11)_2_ heterotetramer plays a role in the subcellular distribution of early and recycling endosomes [11], [12]. One might, however, speculate that calcium-dependent membrane association, *e.g.* at the plasma membrane or along the protein recycling pathway, are regulated by p11 binding and heterotetramer formation [8]. Indeed, the p11 light chain appears to be necessary for AnxA2 binding to the plasma membrane and to the cortical actin network [10], both mechanisms being also regulated by the presence of Ca^2+^ [2], [3]. In addition, (AnxA2)_2_-(p11)_2_ association to the plasma membrane seems to be regulated by direct binding of the heterotetramer to phosphatidylinositol (4,5) bisphosphate [34], [35] and p11 itself seems to play a role in the trafficking of some ion channels and receptors — reviewed in [8]. Rescher and Gerke thus recently proposed that p11 tethers some transmembrane proteins to AnxA2, and thereby anchors them at specific membrane sites or helps their transport to the plasma membrane [8]. It is conceivable that specific functions of AnxA2 are differentially regulated at different sites and along different trafficking routes by separate mechanisms.

## Materials and Methods

### Cells, antibodies and reagents

Baby Hamster kidney cells (BHK21) and HeLa cells were grown as previously described [15]. The monoclonal antibody against Rab5 was a gift from R. Jahn (Göttingen, Germany), monoclonal antibodies against AnxA2 (HH7 and H28) and p11/S100A10 (H21), were gifts from V. Gerke (Münster, Germany, [10], [21], [22], [23]). Rabbit polyclonal antibodies against EEA1 (early endosomal antigen 1) and Lamp1 (lysosomal associated membrane protein 1) were from Alexis Biochemical and Affinity Bioreagents respectively. Monoclonal antibody against tubulin was from Sigma. Monoclonal antibody against GFP was from Roche Diagnostics. Peroxidase-conjugated secondary antibodies were from BioRad and Cy2, Cy3 and Cy5-conjugated fluorescent secondary antibodies were from Jackson Immunoresearch. 10,000 Da rhodamin-dextran as fluorescent fluid phase marker was from Molecular Probes. F-actin was labeled with Allexa fluor 486nm coupled phalloidin (Invitrogen).

### Plasmids, recombinant proteins and RNAi

cDNA plasmid coding for human AnxA2A65E -GFP, which is here referred to as AnxA2^H28^ because of the presence of the H28 antibody epitope, was a gift from V. Gerke (Münster, Germany). For recombinant AnxA2 construction, human AnxA2^H28^ cDNA was cloned in pGEX-5X-1 expressing vector (Clontech) and GST-protein was produced in BL21 bacteria strain. The GST tag was removed by Factor Xa cleavage and benzamidine treatment (Amersham). The quality of recombinant protein was checked by SDS-PAGE and Coomassie staining. For p11 downregulation, two 21 nucleotides RNA duplexes (DNA target sequence 1: AAC GGA CCA CAC CAA AAT GCC and DNA target sequence 2: AAT GCC ATC TCA AAT GGA ACA) of the human p11 sequence were obtained from Qiagen-Xeragon; for ctrl siRNA, we used VSV-G (Vesicular Stomatisis Virus, protein G) siRNA. AnxA2 downregulation was obtained by Dicer-generated siRNA duplexes. Briefly, an amplicon of the target sequence (nucleotides 1–320 from human AnxA2 cDNA, PCR oligos for T7-promoter based cloning: TAA TAC GAC TCA CTA TAA GGG AGA ATG TCT ACT GTT CAC G sense and TAA TAC GAC TCA CTA TAG GGA GAC TGA GCA GGT GTC TTC antisense) was transcribed *in vitro* to generate dsRNA; dicing reaction and d-siRNAs purification were done accordingly to the manufacturer's instructions (Invitrogen). HeLa and BHK cells were transfected with Fugene-6 (Roche Diagnostics) 6 h after seeding for cDNA transfection (total time of overexpression: 48 h) and with lipofectamine 2000 (Invitrogen) 24 h after seeding for RNAi experiments (total time for silencing: 72 h).

### Microscopy

Cells cultured on coverslips were fixed with 3% PFA and permeabilized with 0.1% saponin in PBS/serum buffer except when using special permeabilization buffer, before fixation, containing 0.1% Triton 100× in 100 mM KCl, 2 mM MgCl_2_, 1 mM CaCl_2_ and 1 mM Hepes pH 6.9. Pictures were captured with a Zeiss Axiophot microscope equipped with a 63× Plan-Neofluar objective or with a Zeiss LSM 510 META confocal microscope equipped with a 63× Plan-Apochromat objective and HeNe1, HeNe2 and Argon lasers. To quantify the colocalization between EEA1 and AnxA2-GFP in mock- and p11 RNAi-treated cells, we counted the number of EEA1-positive structures that also contained AnxA2-GFP per 100 µm^2 ^in each focal plane. Similarly, the number of vesicles containing Lamp1 and rhodamin-dextran (after pulse-chase internalization as above) after mock-, p11, or AnxA2-RNAi was quantified by counting the number of vesicles containing both markers per 100 µm^2 ^in each focal plane.

### In vivo endocytic transport assay

Cells were grown on coverslips and then incubated with 3 mg/mL rhodamin-dextran for 10 min at 37°C in GMEM containing 10 mM Hepes medium. Alternatively, cells were re-incubated in marker-free medium for an additional 40 min at 37°C. Then, cells were fixed and processed for immunofluorescence, as above.

### Subcellular fractionation

Early and late endosomes were purified as previously described [20] from BHK cells. A total endosomal fraction was prepared from HeLa cells, as described [24]. The analysis of total membranes and cytosol was carried out by high-speed centrifugation of post-nuclear supernatants at 70.000 rpm for 30 min. Membranes and cytosol were recovered from the high-speed pellet (HSP) and high-speed supernatant (HSS), respectively.

### Annexin A2 binding to liposomes and endosomes

5 µg of purified recombinant AnxA2 (AnxA2^H28^) were incubated for 30 min at 37°C with liposomes containing phosphatidic acid (PA):phosphatidylethanolamine (PE):cholesterol, (2∶2∶1) or with purified early endosomes in 50 mM Hepes, 1mM EGTA (pH 7.4), 100 mM KCl buffer. Binding was analyzed after separation of membrane-bound and free AnxA2 by ultracentrifugation at 35.000 rpm for 60 min at 4°C. Lipids used for liposomes preparation (PA, PE and cholesterol) were from Sigma.

### Biochemical analysis

Total cell lysates were prepared using TNE buffer (20 mM tris pH 7.4, 150 mM NaCl, 1 mM EDTA), and proteases inhibitors (aprotinin, leupeptin and pepstatin) and 1% NP40. For immunoprecipitation assays, this lysis buffer was supplemented with 10% glycerol. Briefly, cell extracts from transfected BHK or HeLa cells were diluted with TNE buffer to a final NP40 concentration of 1%, complemented with 4 µg anti-GFP antibody and proteinA-Sepharose beads (Amersham), and incubated for 2 h at 4°C. Beads were then washed with TNE buffer with 10% glycerol and resuspended in Laemmli buffer [36]. Lysates and immunoprecipitates were analyzed by SDS-PAGE, using 12% acrylamide gels, and western blotting. For p11 western blotting analysis, PVDF (Millipore) membranes were used. Western blotting was carried out using the SuperSignal® West Pico chemiluminescent substrate (Pierce Chemical Co); exposure times were always within the linear range of detection. Chemiluminescent signal was quantified with ImageJ software (NIH).
